# Mortality in Stray Kittens under Eight Weeks Old: Focusing on Congenital Malformations

**DOI:** 10.3390/vetsci11100461

**Published:** 2024-10-01

**Authors:** Gael Contreras, Carlos Viegas, Adelina Gama, Filipe Silva, Isabel Pires

**Affiliations:** 1 Department of Veterinary Sciences, University of Trás-os-Montes e Alto Douro, 5000-801 Vila Real, Portugal; gaelcontrerasfdez@gmail.com (G.C.); cviegas@utad.pt (C.V.); agama@utad.pt (A.G.); fsilva@utad.pt (F.S.); 2 Animal and Veterinary Research Center (CECAV), University of Trás-os-Montes e Alto Douro, 5000-801 Vila Real, Portugal; 3 Associate Laboratory for Animal and Veterinary Sciences (AL4AnimalS), 5000-801 Vila Real, Portugal

**Keywords:** cat, fading kitten syndrome, mortality, feline neonatology

## Abstract

**Simple Summary:**

Neonatal and pediatric diseases are complex and can cause high mortality in kittens, associated with bacterial infections, blood type mismatches, congenital defects, viral and parasitic diseases, and poor care, among others. This study aimed to identify the leading causes of death and prevalence of congenital malformations by performing necropsies on 68 stray kittens under two months old. Results showed that respiratory lesions were the leading cause of death in the youngest kittens, while older kittens primarily suffered from gastrointestinal problems. Infectious diseases were common in all age groups, and 40% of the kittens had congenital malformations, with the most common being megaesophagus, cardiovascular anomalies, and bone and kidney defects. The findings highlight the importance of good hygiene in preventing infections and emphasize the need for better care and preventive measures to enhance kitten survival rates.

**Abstract:**

Neonatal and pediatric mortality in kittens could be associated with bacterial infections, complications from inadequate management, congenital malformations, neonatal isoerythrolysis, parasitic diseases, and viral diseases. The complexity of causes, coupled with kittens’ physiological and immunological immaturity, complicates the diagnosis and treatment of disease, highlighting the necessity for preventive measures. This study aimed to identify the leading causes of death and the occurrence of congenital malformations in stray kittens. Necropsies were performed on 68 kittens, all aged under two months. Results indicated that respiratory lesions were the leading cause of death in the youngest group, while gastrointestinal problems were more prevalent in older groups. Infectious causes were predominant across all age groups. Congenital malformations were observed in 40% of the animals, with megaesophagus, cardiovascular anomalies, bone defects, and kidney defects being the most prevalent. The findings underscore the critical importance of hygiene in preventing infections and related complications. Promoting sterilization and sanitary control in stray cats is essential to reduce overpopulation and improve living conditions.

## 1. Introduction

The exact number of cats worldwide is unknown, but over 200 million cats are estimated to live as pets, with millions more living on the streets. The lack of reliable records on the populations of intact and sterilized cats complicates the estimation of the number of animals born each year and calculating fetal and neonatal mortality rates [1,2].

Due to cats’ high reproductive rate [3,4,5], some authors emphasize the importance of sterilizing stray animals to control birth rates and reduce feline overpopulation [6]. It is estimated that more than 75% of kittens born to stray cats die or disappear within the first six months, reflecting the poor living conditions of these animals without reproductive control [7,8,9,10].

Kitten mortality during the first two months of life is a critical issue impacting animal welfare and survival. This period is particularly delicate, with various causes contributing to significant mortality rates, requiring special attention to improve the survival chances of newborns [11,12,13].

Key factors determining a kitten’s viability and proper development include adequate colostrum intake within the first 24 h of life, birth weight, and environmental factors like hygiene, temperature, humidity, appropriate nutrition, and litter size [13,14,15,16].

Kittens with low birth weight may have a deficit in pulmonary surfactant production, leading to significant lung issues [17]. Environmental conditions are crucial because kittens are poikilothermic until they reach four weeks, necessitating carefully regulated temperatures and humidity levels (55%). During their first week of life, kittens should be kept in an environment with temperatures ranging from 32 to 34 °C (89.5 to 93 °F) up to 1 week of age. This temperature can be gradually reduced to 24 °C–27 °C by 3 weeks of age as they age over the following weeks [12]. Failure to meet these conditions can result in dehydration, the inhibition of the sucking reflex, and development of paralytic ileus, leading to intestinal content retention and the potential reabsorption of toxins, causing endotoxemia and septicemia [17,18]. Good hygiene and adherence to deworming and vaccination protocols are also essential in preventing bacterial septicemia, viruses, or parasitoses within neonatal and pediatric phases [17,18,19,20].

Adequate nutrition is also essential. A kitten must consume between 20 and 26 kcal per 100 g of body weight daily, aiming for a weight increase of 10% daily during the first week of life. It is important to ensure that the maximum stomach capacity (5 mL per 100 g of body weight) is not exceeded to avoid overhydration, colic, and aspiration pneumonia [17,18]. Colostrum intake is vital for the organic maturation of kittens, enabling more efficient nutritional absorption and weight gain. Due to its immunomodulatory and antimicrobial properties and its effects on the cardiovascular system, failure to ingest colostrum in a timely manner can lead to bradycardia, hypoxia, and electrolyte and acid–base imbalances such as metabolic acidosis, and even fatal necrotizing enterocolitis [21,22,23].

The viability of kittens born to free-roaming cats is even more challenging than domestic cats. A lack of veterinary care, uncontrolled breeding, insufficient disease prevention in kittens and mothers, inadequate breeding management protocols, poor nutrition, and the absence of temperature- and light-controlled environments lead to significantly higher mortality rates than kittens raised by owners [7]. On the other hand, behaviors observed in certain free-roaming or feral cat colonies, such as communal nesting [24,25], may contribute to the high mortality of kittens [26].

The percentage of stray kittens that die within 6 to 8 weeks of age ranges from 12.8% to 48%, with some studies indicating that up to 90% do not survive beyond 6 months. These rates are higher than those reported for pedigree breeding cats, around 17–28% [7]. Therefore, our study aims to identify the primary causes of mortality in kittens under two months old born on the streets and taken into shelters in Northern Spain. Additionally, it seeks to evaluate the prevalence of congenital malformations in this population.

## 2. Materials and Methods

### 2.1. Sample Collection and Post-Mortem Examination [27]

This study included 68 kittens aged under 8 weeks old. The animals, either found alone or abandoned, had previously been collected alive from the streets by official entities from two cities and transported to shelters in the north of Spain to receive care. In some cases, support was also provided by foster families. In the four shelters included in this study, over one year, the animals that did not survive were subjected to necropsy for diagnostic purposes. They were kept refrigerated and necropsied within 24 h. If this was not possible, the corpses were immediately frozen and then thawed 6–12 h before the necropsy. All animals underwent a systematic and thorough post-mortem examination (G.C. and I.P.) to ensure consistency in the procedure and minimize variability in the findings. The necropsy technique was adapted from Löhr et al. [28].

The following organs were systematically collected from all animals, the brain, pre-scapular lymph node, thymus, lung, heart, liver, spleen, stomach, intestine, and kidney, regardless of lesions and the corpse’s preservation method. Samples were fixed in 10% buffered formalin, processed, embedded in paraffin, and stained with hematoxylin and eosin for microscopic observation in the Histology and Pathology Laboratory of the University of Trás-os-Montes e Alto Douro according to conventional methodology. The histopathological analyses were performed at the Histology and Anatomical Pathology Laboratory of the University of Trás-os-Montes and Alto Douro.

The age, breed, gender, and clinical history of each animal were also recorded, as well as any examinations conducted during life. Only animals aged two months or less were included, and the age of each animal was categorized as less than 1 week, between 1 and 4 weeks, and between 4 and 8 weeks [29].

### 2.2. Morphological Diagnosis

The morphological diagnosis for each organ was based on the lesions observed during the necropsy, the macroscopic diagnosis, and the lesions identified in the microscopic examination.

To specifically analyze congenital abnormalities, a separate category was created to indicate the presence or absence of congenital alterations and another category to classify the different malformation types.

### 2.3. Cause of Death

The primary cause of death was classified for each animal according to the criteria established by Kent et al. [30]. This classification was based on identifying the main affected organ system and the underlying pathophysiological process. Two researchers with experience in pathology (I.P.) and internal medicine (C.V.) categorized each case simultaneously. In cases of no agreement, the group was expanded to include two additional researchers with expertise in both areas (A.G. and F.S., respectively). In cases where the primary cause of death could not be definitively established, it was recorded as undetermined [30].

Death causes were categorized based on the affected systems: cardiovascular, dermatologic, endocrine, gastrointestinal, genital, hematopoietic, hepato-biliary, musculoskeletal, neurologic, ophthalmologic, respiratory, urological, multi-organ/systemic, and undetermined. The multisystemic category included cases where multiple systems were affected [30].

Deaths were also classified into 10 pathophysiological categories: congenital, degenerative, infectious, inflammatory, ischemic, metabolic, neoplastic, toxic, traumatic, and vascular. In cases where the pathophysiological process could not be determined, it was recorded as indeterminate [30,31].

The etiological diagnosis was not made due to limitations in performing complementary tests. Even so, when clinical information and complementary tests, such as those for feline panleukopenia virus or feline infectious peritonitis, were available, their results were considered for the diagnosis. In our study, only 25 cases underwent testing for the described diseases (20 for feline panleukopenia virus and 5 for feline infectious peritonitis).

### 2.4. Statistical Analysis

All data were recorded in an Excel file. The S.P.S.S. program (version 23.0; IBM SPSS Inc., Chicago, IL, USA.) was used for the statistical analysis. Results were expressed in absolute and relative frequency. Associations between lesions and causes of death and the age and the sex of the animals were evaluated using the Chi-square test (χ^2^). Associations with a *p*-value < 0.05 were considered statistically significant.

## 3. Results

### 3.1. Sample Description

Of the 68 necropsied animals (26 females and 42 males), 23.5% were less than 1 week old, 44% were between 1 and 4 weeks old, and 32% were between 1 and 2 months old. In the group of less than one week old kittens, 44% were female, and 56% were male. The distribution was similar for kittens older than one week, with 43% female and 56.7% male. In kittens over four weeks old, 73% were male and 27% female. These free-roaming kittens became orphans, either due to the death of their mother or abandonment.

### 3.2. Morphological Diagnosis of Lesions

**Figure 1:**
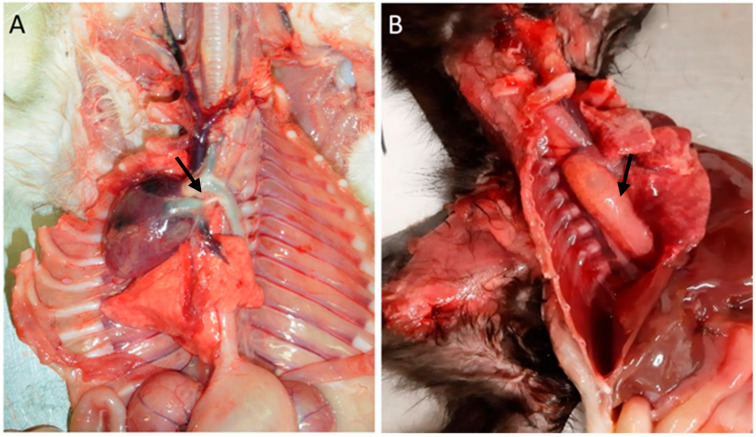
Cardiovascular congenital lesions. (**A**) Persistent ductus arteriosus (arrow). (**B**) Persistent right fourth aortic arch and megaesophagus (arrow).

Among cardiovascular anomalies, 11 (16%) kittens presented lesions, including a ventricular septal defect (*n* = 1), congestion (*n* = 1), persistence of the right fourth aortic arch (*n* = 7), persistent ductus arteriosus, and persistence of the right fourth aortic arch (*n* = 1; also with), Figure 1. No significant macroscopic and microscopic lesions were observed in 57 animals.

In the respiratory system, 33 animals (49%) presented alterations, with bronchopneumonia being the most frequent in 17 animals, followed by edema in 6 animals, and other conditions such as congestion in 2 animals, emphysema in 2 animals, and hemorrhage in 2 animals. In three animals, the gross (food content in the trachea and bronchi) and microscopical lesions allowed for diagnosing aspiration pneumonia.

**Figure 2:**
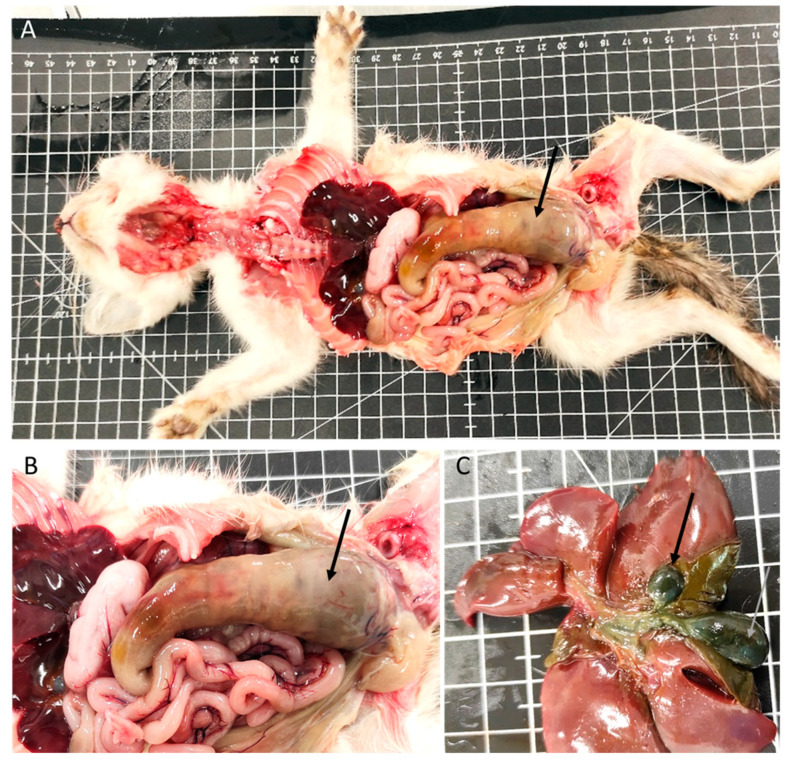
Digestive system and liver congenital lesions. (**A**,**B**) Megacolon (arrow). (**C**) Gallbladder congenital lesions: duplication (arrows).

In the gastrointestinal system, 56% of the animals had lesions (38 animals). Megaesophagus was identified in eight animals (Figure 1B), one of which had a hemorrhagic infarction of the esophagus. Gastric lesions occurred in 16% of the cases, including gastric dilatation (seven cases), ulceration (three cases), and the presence of a foreign body (one case). Of the 36 animals with enteric pathology, nonspecific enteritis was the most common finding, observed in 27 animals. Foreign bodies were present in one animal, and parasitic enteritis was found in four animals: three cases caused by nematodes (*Toxocara cati*) and one case by a tapeworm (*Dipylidium caninum*). Fibrinous enteritis was observed in three animals, and megacolon (Figure 2A,B) was detected in one animal, associated with stricture of the distal colon.

The liver exhibited macroscopic and/or microscopic alterations in 40 kittens (59%). Among these, 26 had hepatic congestion, 6 had embolic hepatitis (1 with omphalophlebitis lesions), and 3 animals had lesions compatible with perihepatitis. Additionally, three animals presented gallbladder duplication (Figure 2C).

Also, 22% of the kittens had spleen lesions, 21% exhibited white pulp hyperplasia, and 2.5% had reactive lymph nodes. Seven animals presented with purulent peritonitis.

**Figure 3:**
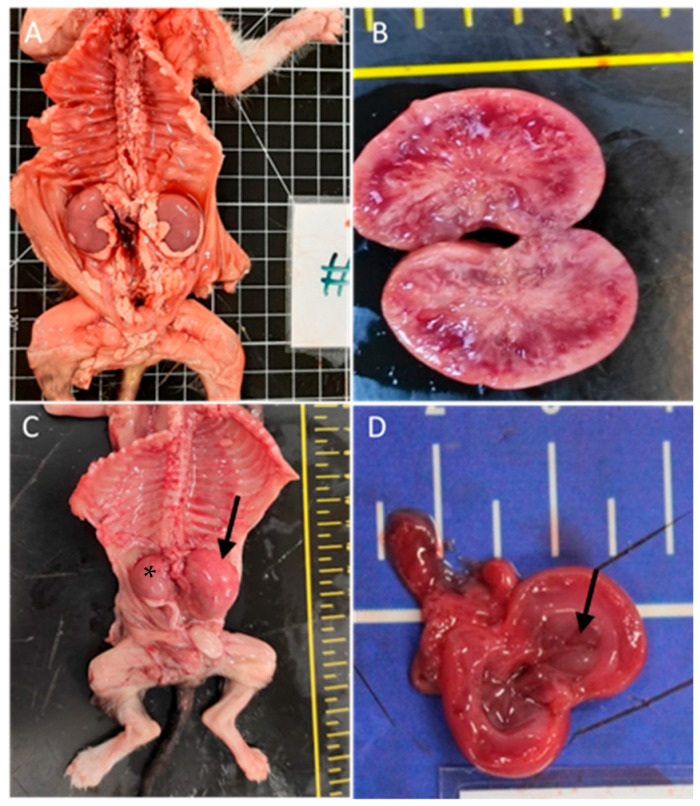
Renal congenital lesions. (**A**) Bilateral nephromegaly due to pelvis dilation. (**B**) Kidney dysplasia. (**C**) Hypoplasia of the right kidney (asterisk) and compensatory hyperplasia of the contralateral kidney (arrow). (**D**) Pelvis dilatation (arrow).

The urinary system was affected in 31% of the cases (*n* = 21), predominantly with renal congestion (10 animals). Renal hypoplasia was noted in three animals, being unilateral with associated compensatory hypertrophy in two of them; pelvic dilation in three animals; cystitis in two animals; renal dysplasia in two cases with embolic nephritis found in one animal; and embolic nephritis alone was detected in one animal and one case of persistent kidney fetal lobulation. Figure 3 shows renal congenital lesions.

**Figure 4:**
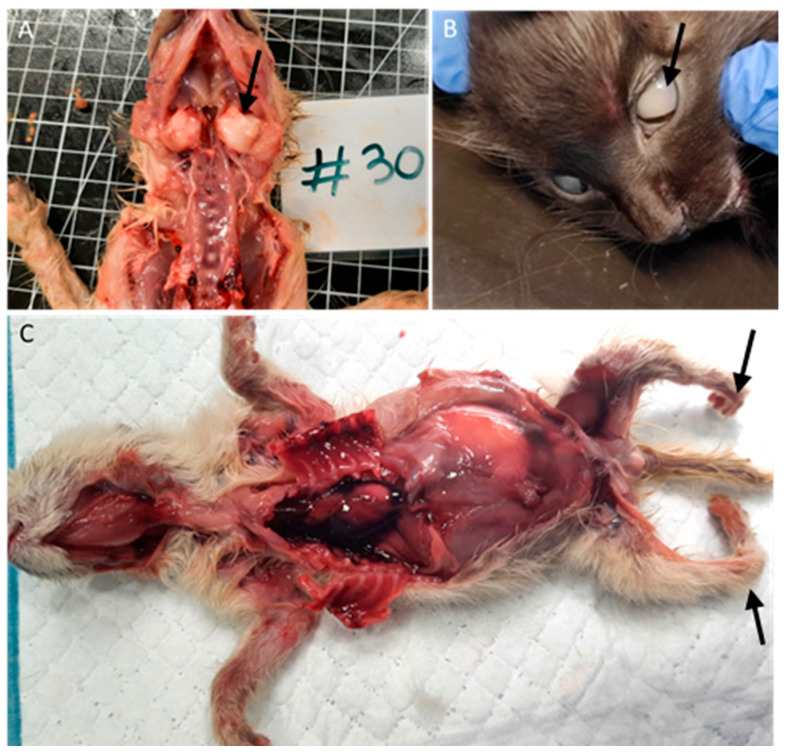
Bone and ocular congenital lesions. (**A**) Tympanic bullae malformations (arrow). (**B**) Congenital cataract (arrow). (**C**) Atrogryposis (arrow).

Among the nine animals with bone malformations in the skeletal system, seven had tympanic bullae malformations with enlargement (Figure 4A), and two had limb malformations (peromelia in one case and arthrogryposis), Figure 4C.

Two animals (3%), one with conjunctivitis and one with congenital cataracts (Figure 4B), had ocular lesions, and three animals (4.5%) had thymic congestion and hemorrhage.

**Figure 5:**
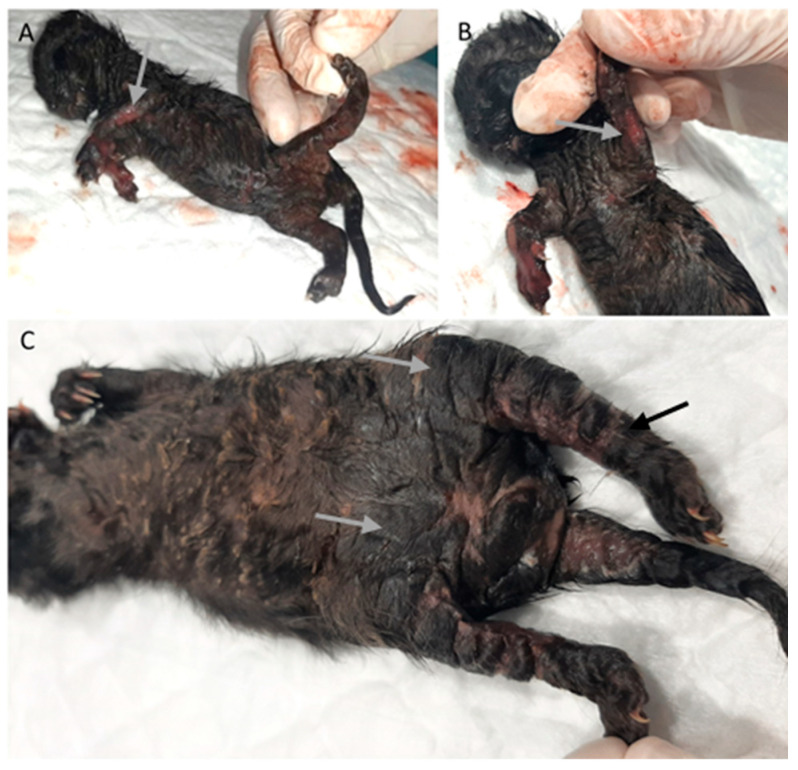
Skin congenital lesions in two kittens from the same litter. (**A**,**B**) Epitheliogenesis imperfecta (arrow). (**C**) Peromelia (black arrow) and congenital ichthyosis (gray arrow).

Four animals (6%) had skin lesions, three congenital, including epitheliogenesis imperfecta (*n* = 1) and ichthyosis (*n* = 2), Figure 5.

As described, this study revealed a variety of congenital malformations (Figure 1, Figure 2, Figure 3, Figure 4 and Figure 5). Overall, 40% of the animals had congenital malformations. Of these, 14 animals (52%) had a single malformation, while 13 (48%) had multiple malformations. These results are summarized in Table 1.

Among the types of malformations observed, renal malformations were the most frequent, detected in nine animals (33% of total malformations): three animals (11%) had renal hypoplasia, three animals (11%) had renal pelvis dilation, and two animals (7.4%) had renal dysplasia. Tympanic bulla malformation occurred in seven animals (26%). Megaesophagus was also observed in eight animals (30%), all with a persistent right aortic arch. Cutaneous malformations, specifically ichthyosis, were observed in two animals (7.4%), with one also presenting with arthrogryposis. Other isolated malformations included arthrogryposis in one animal (3.7%), epitheliogenesis imperfecta in one animal (3.7%), hydrocephalus in one animal (3.7%), and megacolon in one animal (3.7%).

Other multiple malformations were also recorded. Two animals (7.4%) presented tympanic bulla malformation and a duplicated gallbladder. Multiple cardiac congenital defects were observed in two animals (7.4%) that presented a combination of a persistent right fourth aortic arch alongside a ventricular septal defect (one case) or ductus arteriosus persistence (one case). One animal (3.7%) had a combination of ichthyosis, arthrogryposis, and peromelia. One animal (3.7%) had megaesophagus with multiple associated conditions, including agenesis of the spleen, renal pelvis dilation, and cataracts.

### 3.3. Cause of Death

Considering the affected organ systems, it was observed that in 32% of the cats, the cause of death was attributed to gastrointestinal diseases, 28% to respiratory diseases, 19% to multi-organ or systemic conditions, 4.4% to skin diseases, 4.4% to urinary diseases, and 1.5% to cardiovascular diseases. In 10% of the cases, the cause of death could not be defined and was classified as indeterminate, as described in Table 2.

The primary causes of death in the studied kittens were categorized based on their pathophysiology (Table 3). Congenital causes accounted for 15% of the deaths, indicating that these kittens had inherent defects or malformations incompatible with life. Indeterminate causes represented 10% of the cases. Infectious causes were the most prevalent, comprising 66% of the deaths. In 25 cases, complementary diagnostic tests were performed (20 tests for feline panleukopenia and 5 tests for feline infectious peritonitis). The tests confirmed a viral etiology in only five cases of feline panleukopenia. Inflammatory causes were rare, representing only 1.5% of the cases, while parasitic infections contributed to 5.9% of the deaths. Traumatic causes, such as physical injuries, were also rare, accounting for 1.5% of the cases.

As described in Table 3, death was attributed to malformation in 15% of the cases. The malformations observed in these 10 animals included a ventricular septal defect and bone malformation of the tympanic bulla; renal dysplasia; epitheliogenesis imperfecta; renal hypoplasia; ichthyosis (observed in two animals, one of which also had arthrogryposis); megacolon; megaesophagus and splenic agenesis; and a persistent aortic arch, pelvis dilation, and cataracts, as well as megaesophagus, a persistent right fourth aortic arch, and a duplicated gallbladder.

### 3.4. Distribution of Morphological Diagnosis and Causes of Death by Age Class

No statistically significant differences were noted among the morphological diagnoses at different ages. However, the results show some variations in expression between age groups.

All cardiac lesions were observed in animals older than 1 week. The highest incidence of pulmonary alterations was found in animals aged over 1 week up to 4 weeks (60%). Overall, bronchopneumonia was the most common respiratory condition observed across all age groups in our study, but its prevalence decreased in animals older than 4 weeks.

Gastrointestinal lesions were more frequent in animals older than 1 week, with the highest incidence seen in animals aged between 1 and 4 weeks (18 cases), followed by animals older than 4 weeks (14 cases). Gastric lesions were generally most common in animals aged between 1 and 4 weeks. Across all age groups, the most frequent enteric lesion was nonspecific enteritis. Parasitic enteritis was primarily observed in animals older than 4 weeks (three out of four cases). The five cases of feline panleukopenia with a positive test occurred in animals older than 1 week, with three of them in animals over 4 weeks old.

Hepatic lesions overall are more frequent in animals aged 1–4 weeks, except embolic hepatitis, which is more frequent in animals less than 1 week old. Spleen lesions were most frequent in animals aged between 1 and 4 weeks, with half of the cases of white pulp hyperplasia occurring in this age group. Lymph node reactivity was more common in animals older than 4 weeks.

In the kidneys, lesions were most frequent in animals aged 1 to 4 weeks, with only 5 (3 congestion and 1 hypoplasia) out of 21 cases occurring in animals less than 1 week old. Pelvic dilation was observed in animals aged 1 to 4 weeks.

Congenital malformations were more frequent in animals aged between 1 and 4 weeks (12 out of 27 cases). Cardiac congenital lesions were most frequently observed in animals between 1 and 4 weeks of age, with only three out of the eight cases of aortic arch persistence found in animals older than 4 weeks. In the bones, limb malformations occurred in animals younger than 1 week. In comparison, tympanic bull malformations were exclusively found in animals older than 1 week, with half of the cases in those aged between 1 and 4 weeks and the other half in animals older than 4 weeks. The six malformations in animals younger than 1 week included all cases of cutaneous malformations, arthrogryposis, and hydrocephalus, and one case of renal bilateral hypoplasia. In animals older than 4 weeks, malformations involved renal anomalies (one case of renal pelvic dilation and one case of renal dysplasia), megacolon, megaesophagus, a duplicated gallbladder, aortic arch anomalies, and a malformation of the tympanic bulla.

**Figure 6:**
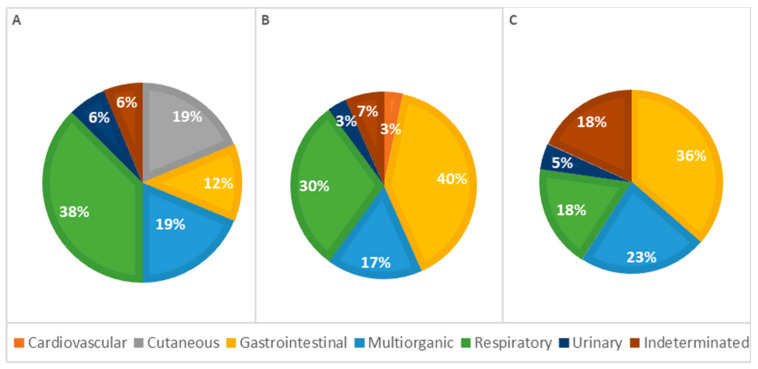
Distribution of causes of death categorized by systems among age classes—(**A**): <1 week; (**B**): 1–4 weeks; (**C**): >4 weeks (*p* = 0.137).

The analysis of the causes of death by age classes revealed differences; however, the statistical analysis showed no significant association (*p* = 0.137; Table 2). In animals under 1 week old, respiratory causes were the most frequent cause of death. In contrast, in older animals (more than 1 week old), gastrointestinal causes were the most common. The percentage of deaths due to respiratory causes decreased with age, while deaths due to gastrointestinal diseases increased correspondingly. Additionally, skin-related causes of death were observed exclusively in animals less than 1 week old. Mortality due to cardiovascular lesions and multi-organ conditions occurred more frequently in animals older than 1 week. To provide better visualization, Figure 6 summarizes these results, showing the percentage of lesions calculated as a proportion of the total cases observed in each group.

**Figure 7:**
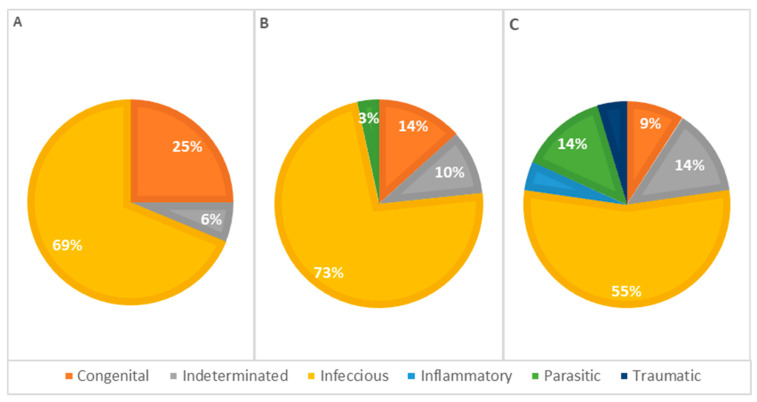
Distribution of causes of death categorized by pathophysiology among age classes—(**A**): <1 week; (**B**): 1–4 weeks; (**C**): >4 weeks (*p* = 0.312).

The statistical analysis of pathophysiological causes of death across different age groups revealed infectious causes as the primary cause at all ages, accounting for 63% of deaths in animals less than 1 week old, 77% in animals aged 1 to 4 weeks, and 55% in animals older than 4 weeks, representing 66% of the total cases. However, no significant statistical association was detected (Table 3). Congenital lesions were more frequent in animals aged 1 to 4 weeks (*n* = 13). Seven animals older than 4 weeks and nine younger than 1 week presented congenital lesions in one or more systems. Deaths due to congenital defects decreased with age, as illustrated in Figure 7.

### 3.5. Distribution of Morphological Diagnosis and Causes of Death by Animal Sex

**Figure 8:**
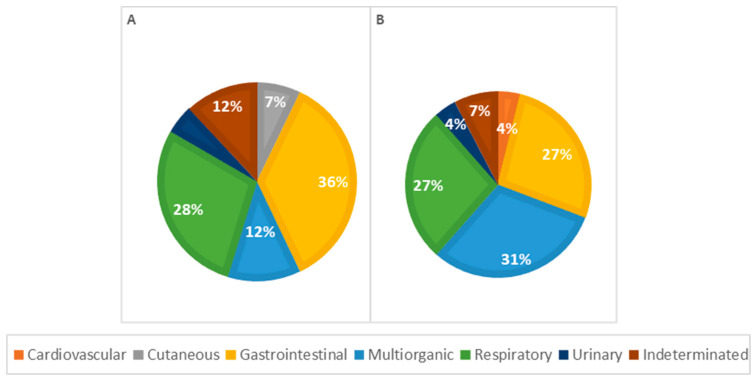
Distribution of causes of death categorized by systems in males (**A**) and females (**B**); *p* = 0.306.

Considering morphological diagnoses, all observed lesions were more frequent in males, except for perihepatitis (67%), hepatic atrophy (100%), pulmonary edema (67%), lymph node reactivity (57%), and cystitis (100%), which were more frequent in females. In general, congenital malformations were more frequent in males (72%), with heart lesions, particularly a persistent aortic arch, and congenital heart defects predominantly seen in males. Renal malformations, such as pelvic dilation and renal dysplasia, were observed exclusively in males, as shown in Figure 8.

Among causes of death in female animals, multi-organ/systemic causes were the most frequent (*n* = 8, 31%), followed by respiratory (*n* = 7, 27%) and gastrointestinal causes (*n* = 7, 27%). In male animals, gastrointestinal causes were the most common (*n* = 15, 40%), with respiratory causes also significantly contributing to mortality (*n* = 12, 28%), as illustrated in Figure 8. However, according to the statistical analysis, no significant association was observed between the cause of death and the animal sex.

**Figure 9:**
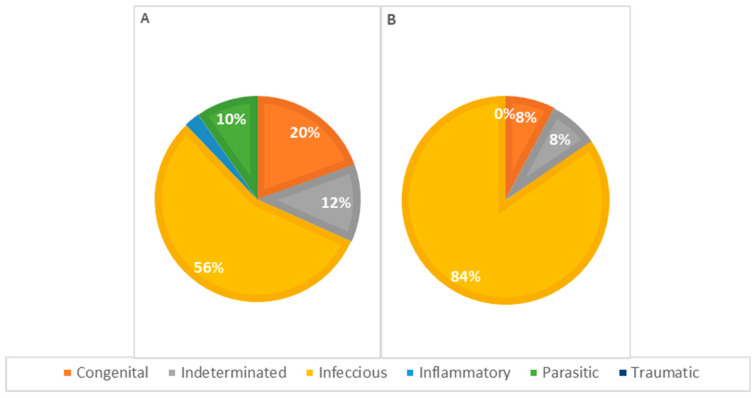
Distribution of causes of death categorized by pathophysiology in males (**A**) and females (**B**); *p* = 0.192.

Concerning the pathophysiology of causes of death across different genders (Figure 9), infectious causes are the primary cause in both females and males. In females, infectious causes accounted for 81% of the cases (*n* = 21), while in males, they accounted for 57% (*n* = 24). Congenital causes were the second most frequent in males, accounting for 19.0% of the cases (*n* = 8), whereas in females, congenital causes accounted for 7.7% (*n* = 2). Indeterminate causes were observed in 7.7% of the cases in females (*n* = 2) and 12% in males (*n* = 5). Parasitic causes were relatively rare, accounting for 3.8% of deaths in females (*n* = 1) and 7.1% in males (*n* = 3). The statistical analysis showed no significant association between the cause of death and the animals’ sex.

## 4. Discussion

Kitten mortality during the first two months of life is a significant issue affecting animal welfare. It represents a significant challenge in neonatal feline health, contributing to high mortality rates due to its multifactorial etiology and the nonspecific and often sudden onset of symptoms [13,32]. Critical factors determining a kitten’s viability and proper development include birth weight, adequate colostrum intake within the first 24 h of life, environmental factors, and appropriate nutrition [18].

Kitten survival, particularly in stray and orphaned animals, presents unique challenges. These animals often face compromised environmental and health conditions exacerbated by the absence of maternal care. So, this study aimed to explore the underlying causes of mortality and the prevalence of congenital malformations in orphaned, free-roaming kittens under two months old.

We observed that nearly half of the kittens died before reaching four weeks of age, reflecting neonatal kittens’ high vulnerability during their first weeks of life. This finding is consistent with the existing literature, highlighting that most neonatal deaths occur before weaning [33,34]. Specifically, several studies have reported that the highest mortality rates occur within the first 2 weeks of life and around the weaning period, typically between 4 and 5 weeks of age [18,32].

To better understand the mortality in the study sample, the post-mortem lesions observed were morphologically classified, which served as a basis for categorizing the causes of death of each animal according to the affected organs and the pathophysiology of the disease.

Regarding morphological diagnoses, nonspecific enteritis was the most frequent enteric lesion across all age groups. In five cases of enteritis, it was possible to diagnose feline panleukopenia (morphologically, classified initially as nonspecific in two cases and fibrinous in three cases) in animals older than 1 week, with three of them being over 4 weeks old. Fibrinous pseudomembranous, characterized by pseudomembrane formation, suggests panleukopenia in cats [34,35]. Parasitic enteritis was also observed in older animals in our study. Other research suggests that parasitic infections can begin soon after birth, with *Toxocara* species often transmitted during the neonatal period. Within the first few days or weeks of life, kittens may contract parasites like *Giardia* spp., *Cryptosporidium* spp., *Coccidia* spp., and *Tritrichomonas fetus* [36]. Embolic hepatitis, which is often associated with omphalophlebitis, is more frequent in animals less than 1 week old. This underscores the importance of maintaining good hygiene practices to prevent bacterial infections, to which these animals are particularly susceptible.

Regarding pulmonary lesions, bronchopneumonia was the most frequently observed respiratory condition across all age groups, although its prevalence decreased in animals older than 4 weeks. This finding aligns with the literature, which suggests that bronchopneumonia, generally caused by bacterial infections, can develop in young or immunocompromised animals. As described in other studies, bronchopneumonia may also occur as a secondary complication following viral infections, such as those caused by herpesvirus-1 or calicivirus [37,38].

Although there were no statistically significant differences between the lesions observed in males and females, it is noteworthy that certain conditions were more frequent in females: perihepatitis, hepatic atrophy, pulmonary edema, lymph node reactivity, and cystitis. On the other hand, renal malformations, such as pelvic dilation and renal dysplasia, were found exclusively in males. Further studies with larger sample sizes are needed to validate these findings and determine whether these trends are consistent across a broader population.

Among the mortality causes, our results are aligned with those of other studies [17,39,40], except for deaths caused by trauma [7], which were rare in our study. Respiratory lesions were the leading cause of death in kittens less than one week old, and in kittens aged one to eight weeks, gastrointestinal problems emerged as the primary cause of death. Among females, multi-organ/systemic causes were the most frequent. Additionally, according to pathophysiology, infectious diseases were common across all age groups, both in males and females, accounting for 66% of the deaths. Indeed, neonatal kittens are highly susceptible to respiratory problems due to their immature lungs and potential lack of adequate colostrum intake, which is essential for immune protection [12]. Also, even if they receive colostrum, the mothers may not have been vaccinated or have immunity from prior infections. In kittens aged one to eight weeks, gastrointestinal diseases increased, possibly due to dietary changes and environmental exposures as kittens begin to wean [17,39,40]. Moreover, bacterial and viral infections are prevalent in neonatal kittens due to their underdeveloped immune systems and the high-density living conditions often found in street colonies and shelters [34].

In our study, viral enteritis (panleukopenia) was more commonly diagnosed in animals older than 4 weeks. In contrast, conditions such as bronchopneumonia, embolic hepatitis, and embolic nephritis—suggestive of bacterial infections—were more frequent in younger animals, particularly those under 1 week old or between 1 and 4 weeks old. Despite the limitations of our study in terms of complementary testing, it is interesting to compare these findings with other studies. Bacterial infections are more prevalent in neonates before weaning, with isolated pathogens including *E. coli*, *Streptococcus canis*, *Staphylococcus aureus*, *Staphylococcus pseudointermedius*, *Klebsiella* spp., *Pseudomonas* spp., *Campylobacter* spp., *Chlamydiae* spp., and anaerobic bacteria. Conversely, viral infections tend to occur post-weaning, as maternally derived antibodies begin to diminish. Infections with calicivirus and feline herpesvirus-1, feline panleukopenia virus, and feline coronavirus (infectious peritonitis virus) are most commonly observed at this stage. However, it should be considered that orphaned kittens or those with insufficient colostrum intake and born to unvaccinated mothers are at a significantly higher risk of developing viral infections earlier in life [32,36].

While an etiological diagnosis for each case would have been ideal for understanding infectious diseases, this was not always feasible. Economic constraints, particularly in shelter medicine, often limit access to complementary diagnostic tests, making identifying the specific pathogens or conditions responsible for the animals’ deaths challenging. Furthermore, most of the animals had been frozen, which hindered microscopic examination by destroying cellular structures and creating tissue artifacts, ultimately compromising the diagnostic process—especially in identifying viral inclusion bodies or cellular degeneration [28,37]. Ideally, bodies should be refrigerated, and necropsies should be performed promptly to preserve tissue integrity and allow for the observation of structures suggestive of inclusion bodies. Therefore, although we cannot draw definitive conclusions about the specific agents involved, we hope this study contributes to a better understanding of the causes of mortality.

The other objective of this study was to determine the frequency and types of congenital abnormalities in stray kittens. Congenital malformations are known to be significant causes of morbidity and mortality in kittens overall [41]. Our study observed congenital malformations in 40% of cases, with a higher frequency in animals aged between 1 and 4 weeks. These findings significantly diverge from the literature, where the reported incidence of congenital malformations in general cat populations is up to 10% [28]. Furthermore, congenital defects are typically more common in purebred cats than mixed-breed cats. In fact, between 5% and 25% of litters in purebred cats are reported to have at least one congenital defect [4]. However, it is acknowledged that many malformations may go undiagnosed. Münnich (2022) suggests that the percentage of kittens with congenital anomalies could be higher than usually reported, especially regarding visceral abnormalities [32]. Additionally, chromosomal abnormalities in kittens, which cause lethal developmental errors, can cause embryonic or fetal death and be diagnosed as subfertility [42,43].

Among the types of malformations observed, the most frequent were renal malformation, followed by bone abnormalities and cardiac disorders, such as a persistent right aortic arch. Rarer cases included cutaneous malformations, specifically ichthyosis, epitheliogenesis imperfecta, hydrocephalus, and megacolon. Some of these conditions are consistent with descriptions in the literature. Common congenital malformations reported in previous studies include a cleft palate, a portosystemic shunt, heart defects, megaesophagus, atresia ani, sternum conformation defects, and diaphragmatic hernias (both peritoneopericardial and peritoneopleural) [37,44,45]. It is worth noting that in 48% of the cases with congenital malformations, these were multiple malformations, a condition sporadically reported in cats [46,47,48,49].

Certain congenital defects can lead to neonatal death, such as hydranencephaly, while others, like unilateral renal agenesis, may be incidental findings [37]. In our study, the congenital malformations observed in under-1-week-old animals included all cutaneous malformations, arthrogryposis, and hydrocephalus. On the other hand, renal malformations, megaesophagus, megacolon, a duplicated gallbladder, aortic arch anomalies, and a malformation of the tympanic bulla were mainly identified in animals older than 4 weeks. As for the cause of death, congenital anomalies accounted for 15% of the mortality, namely a ventricular septal defect, renal dysplasia, epitheliogenesis imperfecta, bilateral renal hypoplasia, ichthyosis, megacolon, and cases involving multiple malformations.

Additionally, we observed casos de arthrogryposis occurring alone or in combination with other abnormalities, such as peromelia and bilateral congenital cataracts. Skeletal abnormalities in the pelvic limbs and bilateral corneal opacity are described to be linked to mucopolysaccharidosis type I, a congenital storage disease associated with neurological conditions. This disease is characterized by enzymatic deficiencies that result in an excessive deposition of certain compounds in organs such as the liver or the brain’s white matter [44].

Congenital defects can stem from various factors. Some occur unpredictably without an identifiable cause, while others manifest as the visible traits of genetic disorders or result from the mother’s exposure to harmful substances during pregnancy. Certain drugs with teratogenic potential, including griseofulvin, corticosteroids, and metronidazole, can increase the risk of congenital defects. Additionally, maternal factors such as hypervitaminosis A, hypervitaminosis D, and malnutrition can contribute to these abnormalities, alongside maternal diseases like feline infectious peritonitis, panleukopenia, and toxoplasmosis [37,44,45,50]. For example, megaesophagus can be linked to infectious causes, such as viral infections and toxoplasmosis [32]. Furthermore, conditions like hydranencephaly [51] and cerebellar hypoplasia [52,53,54] are associated with panleukopenia due to natural infection or vaccination with a modified-live virus vaccine.

Unfortunately, in our study, confirming any specific etiological factors was impossible. Further research is needed to better understand the underlying causes of the high number of congenital malformations observed in stray kittens. Additionally, raising awareness among animal welfare organizations and veterinarians about the importance of necropsy examinations in determining the causes of death in these animals is crucial. Given the costs associated with these procedures, establishing cooperation protocols with educational institutions is essential, benefiting both parties.

Our study highlights the need for comprehensive post-mortem examinations to identify the underlying causes of death and inform preventative strategies for reducing mortality in vulnerable kitten populations.

## 5. Conclusions

In our study, the leading cause of mortality was infectious disease, occurring during the neonatal, pre-weaning, and post-weaning periods, primarily due to respiratory and digestive infections. Respiratory conditions were the most common in kittens under one week old, while gastrointestinal diseases became predominant in those aged one to eight weeks.

This study also identified a high prevalence of single and multiple congenital malformations. Further studies with complementary tests are needed to deepen our understanding of the causes of mortality. By enhancing this knowledge, we can develop targeted strategies to save the fragile lives of neonatal and pediatric kittens, giving them a real chance to grow and thrive.

## Figures and Tables

**Table 1:** Distribution of Congenital Malformations observed in kittens.

Congenital Malformations	*n*	%
Persistent right fourth aortic arch and megaesophagus	3	4.4
Persistent right fourth aortic arch, megaesophagus, and persistent ductus arteriosus	1	1.5
Persistent right fourth aortic arch, megaesophagus, and duplicated gallbladder	1	1.5
Persistent right fourth aortic arch, megaesophagus, agenesis of the spleen, renal pelvis dilation, and cataract	1	1.5
Persistent right fourth aortic arch, megaesophagus, tympanic bulla malformation	2	2.9
Ventricular septal defect and malformation of tympanic bulla	1	1.5
Megacolon	1	1.5
Renal hypoplasia	3	4.4
Renal dysplasia	2	2.9
Renal pelvis dilation	2	2.9
Hydrocephalus	1	1.5
Tympanic bulla malformation	1	2.9
Tympanic bulla malformation, duplicated gallbladder	2	2.9
Tympanic bulla malformation, persistent kidney fetal lobulation	1	1.5
Arthrogryposis	2	1.5
Arthrogryposis, peromelia, and ichthyosis	1	1.5
Ichthyosis	1	1.5
Epitheliogenesis imperfecta	1	1.5
Total	27	39.7%

**Table 2:** Cause of death categorized by systems among age classes.

Cause of Death—System	<1 Week	1–4 Weeks	4–8 Weeks	*n* (%)
Gastrointestinal	2 (2.9%)	12 (17.6%)	8 (11.8%)	22 (32.4%)
Respiratory	6 (8.8%)	9 (13.2%)	4 (5.9%)	19 (27.9%)
Multi-organ/systemic	3 (4.4%)	5 (7.4%)	5 (7.4%)	13 (19.1%)
Indeterminate	1 (1.5%)	2 (2.9%)	4 (5.9%)	7 (10.3%)
Cutaneous	3 (4.4%)	0 (0%)	0 (0%)	3 (4.4%)
Urinary	1 (1.5%)	1 (1.5%)	1 (1.5%)	3 (4.4%)
Cardiovascular	0 (0%)	1 (1.5%)	0 (0%)	1 (1.5%)
Total	16 (23.5%)	30 (44.1%)	22 (32.4%)	68 (100%)

**Table 3:** Cause of death categorized by its pathophysiology among age classes.

Cause of Death— Pathophysiology	<1 Week	1–4 Weeks	4–8 Weeks	*n* (%)
Inflammatory	0 (0.0%)	0 (0.0%)	1 (1.5%)	1 (1.5%)
Traumatic	0 (0.0%)	0 (0.0%)	1 (1.5%)	1 (1.5%)
Parasitic	0 (0%)	1 (1.5%)	3 (4.4%)	4 (5.9%)
Indeterminate	1 (1.5%)	3 (4.4%)	3 (4.4%)	7 (10.3%)
Congenital	4 (5.9%)	4 (5.9%)	2 (2.9%)	10 (14.7%)
Infectious	11 (16.2%)	22 (33.4%)	12 (17.6%)	45 (66.2%)
Total	16 (23.5%)	30 (44.1%)	22 (32.4%)	68 (100%)

## Data Availability

Dataset available on request from the authors.
